# Proteomic analysis of peritoneal fluid identified COMP and TGFBI as new candidate biomarkers for endometriosis

**DOI:** 10.1038/s41598-021-00299-2

**Published:** 2021-10-22

**Authors:** V. Janša, T. Klančič, M. Pušić, M. Klein, E. Vrtačnik Bokal, H. Ban Frangež, T. Lanišnik Rižner

**Affiliations:** 1 Department of Obstetrics and Gynecology, University Medical Centre Ljubljana, Šlajmerjeva 3, 1000 Ljubljana, Slovenia; 2 Faculty of Medicine, Institute of Biochemistry, University of Ljubljana, Vrazov trg 2, 1000 Ljubljana, Slovenia; 3 Sciomics GmbH, Karl-Landsteiner-Straße 6, 69151 Neckargemünd, Germany; 4 Faculty of Medicine, University of Ljubljana, Vrazov trg 2, 1000 Ljubljana, Slovenia

**Keywords:** Biochemistry, Biomarkers, Molecular medicine

## Abstract

Endometriosis is a common non-malignant gynecological disease that significantly compromises fertility and quality of life of the majority of patients. The gold standard for diagnosis is visual inspection of the pelvic organs by surgical laparoscopy and there are no biomarkers that would allow non-invasive diagnosis. The pathogenesis of endometriosis is not completely understood, thus analysis of peritoneal fluid might contribute in this respect. Our prospective case–control study included 58 patients undergoing laparoscopy due to infertility, 32 patients with peritoneal endometriosis (cases) and 26 patients with unexplained primary infertility (controls). Discovery proteomics using antibody microarrays that covered 1360 proteins identified 16 proteins with different levels in cases *versus* the control patients. The validation using an ELISA approach confirmed significant differences in the levels of cartilage oligomeric matrix protein (COMP) and transforming growth factor-β-induced protein ig-h3 (TGFBI) and nonsignificant differences in angiotensinogen (AGT). A classification model based on a linear support vector machine revealed AUC of > 0.83, sensitivity of 0.81 and specificity of 1.00. Differentially expressed proteins represent candidates for diagnostic and prognostic biomarkers or drug targets. Our findings have brought new knowledge that will be helpful in the understanding of the pathophysiology of endometriosis and warrant further studies in blood samples.

## Introduction

Endometriosis is a common non-malignant gynecological disease with an estimated prevalence of 10% worldwide, which increases to 50% for women with infertility or chronic pain^1^. There are multiple theories including retrograde menstruation, venous dissemination, lymphatic dissemination, congenital Mullerian remnants, coelomic metaplasia, and engrafting of bone-marrow stem cells^2^. Despite major efforts, endometriosis still remains a disease that is not completely understood and that has a poorly defined etiology and a complex pathogenesis. To date, its pathogenesis is known to involve degradation of the extracellular matrix, aberrant apoptosis, angiogenesis, enhanced cell adhesion, cell proliferation, increased oxidative stress, inflammation processes, a disturbed immune system, and more^3–6^. As a result of these pathophysiological processes, endometrial cells survive and proliferate at ectopic sites, which can evoke chronic pelvic inflammation^7^.

Endometriosis significantly compromises the quality of life in the majority of women, and it is a major cofactor in infertility. The gold standard for diagnosis is visual inspection of the pelvic organs by surgical laparoscopy. Due to the non-specific symptoms and surgery-associated risks, it can be over 10 years before women are diagnosed and correctly treated^8^.

The peritoneum, retroperitoneum, ovaries, bowel, and other sites represents a ‘local environment’ for endometrial lesions. Ectopic endometrial cells evoke local inflammation, which is mediated by immune cells and their pro-inflammatory products^4,5,9^. The analysis of peritoneal fluid, that bathes the endometriotic lesions might be a key to a better understanding of endometriosis^10^. Revealing of the underlying pathophysiology of endometriosis at the molecular level would be of great clinical importance. As peritoneal fluid has a complex role in the etiopathogenesis of endometriosis, it should serve as a source of new diagnostic or predictive biomarkers, which might also contribute to the identification of new drug targets. The surface of the peritoneal cavity is large, and it allows passive dialysis of substances between the peritoneal fluid and the blood plasma, where the diffusion rates decrease with molecular weight^11–13^. Studies of peritoneal fluid might thus contribute to the identification of blood biomarkers for non-invasive diagnosis of endometriosis.

We used a proteomic approach with a high-content antibody microarray that covers 1360 proteins to search for proteins with significantly different levels in peritoneal fluid samples from women with endometriosis (cases) compared to women with unexplained primary infertility (controls). The aim of the study was to identify potential diagnostic and predictive biomarkers or drug targets and to contribute to a better understanding of the pathophysiology of endometriosis.

## Material and methods

This study was designed as a prospective case–control study. It consisted of a discovery phase (proteomic study: antibody array analysis) and a validation phase (enzyme-linked immunosorbent assay [ELISA] validation).

### Patient selection

**Figure 1:**
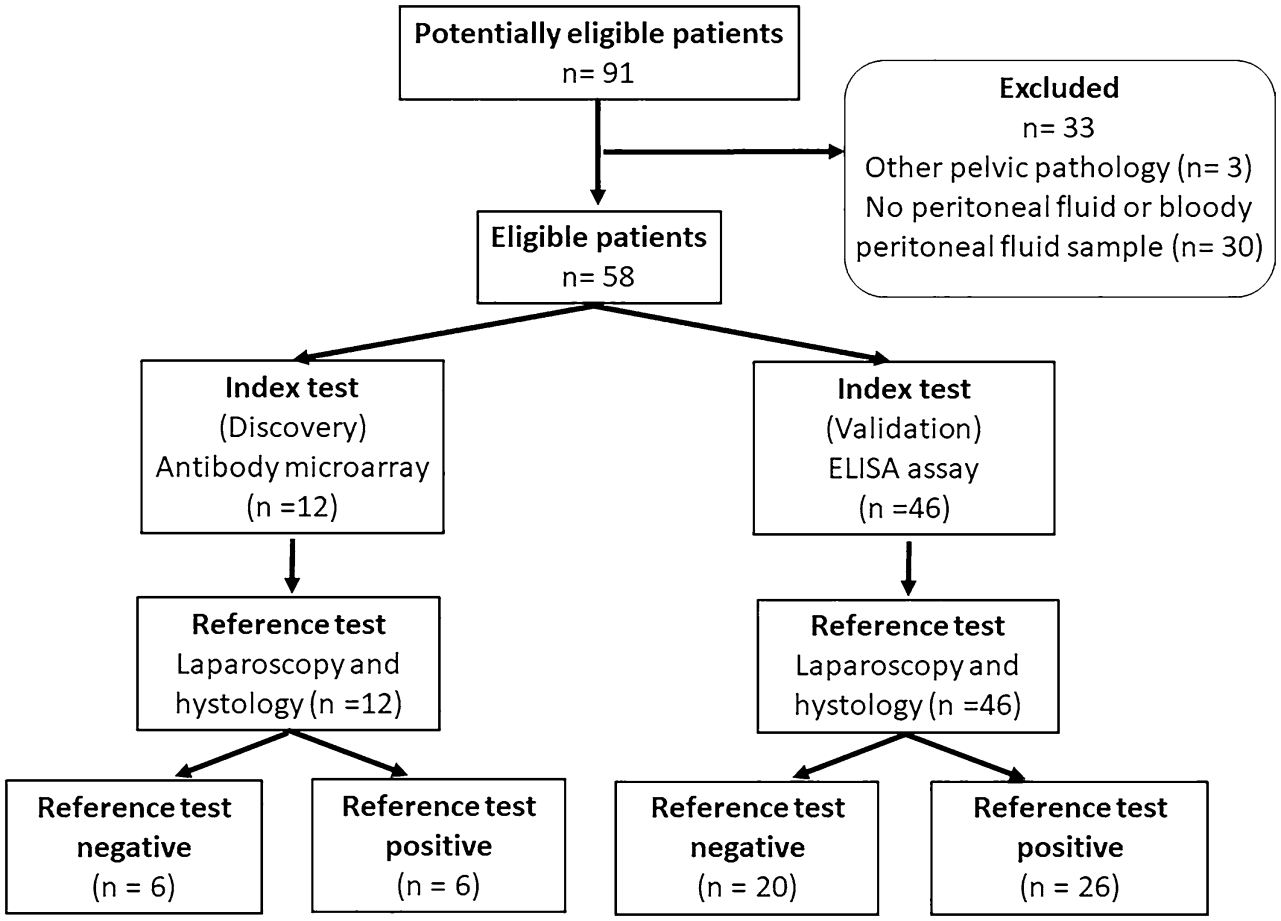
Flowchart of patient recruitment.

The discovery proteomic analysis included a total of 12 women with primary infertility who were stratified according to laparoscopy and histological confirmation (Table 1, Fig. 1). The case group included six women with endometriosis and primary infertility, and the control group included six women with unexplained primary infertility. The validation study with ELISA included 46 women with primary infertility (Table 2, Fig. 1), where the case group included 26 women with endometriosis, and the control group included 20 women with unexplained primary infertility. All of the patients had laparoscopy carried out due to infertility and the diagnosis was confirmed histologically. All of the women had a body mass index (BMI) in the normal range, with a regular menstrual cycle (21–35 days). The partner semen analyses were normal for all of the women included. The further inclusion criteria included: no previous pelvic surgery, no known pelvic inflammatory disease, and ultrasound examination showed no pathology (controls) other than endometriosis (cases). The exclusion criteria included patients who had undergone hormonal therapy in the last year, those with irregular menstrual cycles, and patients with autoimmune diseases, malignant or suspected malignant diseases, previous pelvic inflammatory disease, and leiomyoma uteri or polycystic ovaries. None of the patients had undergone previous pelvic surgery.

**Table 1:** Clinical characteristics of the 12 patients included in the discovery phase.

Parameter	Units	Detail	Controls	Cases	p-value
Total patient numbers	n	–	6	6	–
Mean age (mean ± SD)	years	–	29.6 ± 2.8	28.1 ± 3.1	> 0.05
Mean body mass index (mean ± SD)	kg/m^2^	–	23.8 ± 3.1	23.8 ± 1.7	> 0.05
Menstrual phase	n (%)	Follicular	6 (100)	6 (100)	> 0.05
		Luteal	0 (0)	0 (0)	
Oral contraceptives (last 3 months)	n (%)	No	6 (100)	6 (100)	
		Yes	0 (0)	0 (0)	
Hormonal therapy (last 3 months)	n (%)	No	6 (100)	6 (100)	
		Yes	0 (0)	0 (0)	
Medications (last 1 week)	n (%)	No	6 (100)	6 (0)	
		Yes	0 (0)	0 (0)	
Smoking status	n (%)	Non-smoker	6 (100)	6 (100)	
		Smoker	0 (0)	0 (0)	
		Occasional smoker	0 (0)	0 (0)	
		Former smoker	0 (0)	0 (0)	
Endometriosis	n (%)	Ovarian plus peritoneal	0 (0)	6 (100)	
Revised American Society for Reproductive Medicine score	n (%)	I	0 (0)	0 (0)	
		II	0 (0)	0 (0)	
		III	0 (0)	6 (100)	
		IV	0 (0)	0 (0)

**Table 2:** Clinical characteristics of the 46 patients included in the validation phase.

Parameter	Units	Detail	Controls	Cases	p-value
Total patient numbers	n	–	20	26	–
Mean age (mean ± SD)	years	–	30.5 ± 4.4	29.4 ± 3.7	0.369
Mean body mass index (mean ± SD)	kg/m^2^	–	21.8 ± 1.7	22. 7 ± 3.7	0.754
Menstrual phase	n (%)	Follicular	7 (35)	15 (58)	0.149*
		Luteal	13 (65)	11 (42)	
Oral contraceptives (last 3 months)	n (%)	No	19 (95)	26 (100)	0.4348*
		Yes	1 (5)	0 (0)	
Hormonal therapy (last 3 months)	n (%)	No	19 (95)	26 (100)	0.4348*
		Yes	1 (5)	0 (0)	
Medications (last 1 week)	n (%)	No	17 (85)	21 (80.8)	> 0.999*
		Yes	3(15)	5 (19.2)	
Smoking status	n (%)	Non-smoker	14 (70)	19 (73.1)	0.520**
		Smoker	4 (20)	3 (11.5)	
		Occasional smoker	2 (10)	0 (0)	
		Former smoker	0 (0)	4 (15.4)	
Type of endometriosis	n (%)	Peritoneal	0 (0)	5 (19.2)	
		Ovarian	0 (0)	8 (30.8)	
		Ovarian plus peritoneal	0 (0)	10 (38.4)	
		Deep infiltrating	0 (0)	3 (11.4)	
Revised American Society for Reproductive Medicine score	n (%)	I	0 (0)	7 (26.9)	
		II	0 (0)	0 (0)	
		III	0 (0)	17 (65.4)	
		IV	0 (0)	2 (7.7)	

*Fisher’s exact test.

**Chi-square test for trend.

### Sample and data collection

All of the women who met the inclusion criteria were additionally evaluated. They filled out a questionnaire on their health history, stress levels, use of medications and types of pain (dysmenorrhea, dyspareunia, chronic pain), using a validated visual analogue scale. Stratification was carried out based on the results of the laparoscopy and histological confirmation: the case group only included women with endometriosis and no other pathology, and the control group only included women without any pathology at laparoscopy.

Peritoneal fluid samples were collected during the laparoscopy, before any intra-abdominal procedures were carried out. The pneumoperitoneum was reached using a Veress needle at the umbilicus, and peritoneal fluid was aspirated from the Douglas space using a 2-mm needle. The samples of 3 mL peritoneal fluid were collected in 12-mL plastic tubes (Greiner, Monroe, North Carolina, USA). The tubes were stored at 4 °C. The samples were centrifuged within an hour of collection, at 900×*g* for 5 min at 4 °C. The samples were then aliquoted and stored at − 80 °C. They were used for in-house validation using ELISA assays, or were transported in dry ice to Sciomics GmbH for the proteomics analysis.

### Antibody microarray analysis

The mean bulk protein concentrations of the peritoneal fluid were not significantly different between cases and controls, as 37.92 ± 5.05 mg/mL and 37.53 ± 2.15 mg/mL, respectively (p > 0.05). Twelve samples were labelled for 1 h with scioDyes 1 and then 2 (Sciomics GmbH, Heidelberg, Germany). Excess dye was removed, and the buffer was exchanged to phosphate-buffered saline (PBS) using size exclusion chromoatography. A reference sample was established by pooling identical volumes of all scioDye 2 labelled samples. The 12 scioDye 1 labelled samples were then analyzed in a dual-color approach with a reference-based design using scioDiscover antibody microarrays (Sciomics GmbH, Heidelberg, Germany) that targeted 1360 different proteins with 1830 antibodies^14^. Each antibody was measured on the array by four technical replicate spots. The arrays were blocked with scioBlock (Sciomics GmbH, Heidelberg, Germany) on a microarray hybridisation station (Hybstation 4800; Tecan GmbH, Grödig, Austria), and then incubated for 3 h with identical volumes of sample and reference. The slides were then thoroughly washed (1× PBSTT; 0.1× PBS; water) and dried with nitrogen. Slide scanning was conducted using a microarray scanner (Powerscanner; Tecan, GmbH, Grödig, Austria) with identical instrument laser power and photomultiplier settings.

### Statistical analyses of protein microarray data

Spot segmentation was performed with the GenePix Pro 6.0 software (Molecular Devices, Union City, CA, USA). The raw data acquired were analyzed using the *linear models for microarray data* (*limma*) package of R-Bioconductor after up-loading the median signal intensities. For normalization, a specialized invariant Lowess method was applied^15^. For analysis of the samples, a one-factorial linear model was fitted with *limma* which provided two-sided t-tests or F-tests based on moderated statistics. All of the p values presented are adjusted for multiple testing by controlling the false discovery rate according to Benjamini and Hochberg. The proteins were defined as differential for ǀlog_2_FCǀ > 0.5 and an adjusted p < 0.05. Differences in the protein levels between the different samples or sample groups are presented as log-fold changes (logFC) calculated as base 2. In such studies, comparing samples (cases) *versus* controls, logFC = 1 means that the sample group had on average a 2^1^ = twofold higher signal than the control; logFC =  − 1 means 2^−1^ = 1/2 of the signal in the sample than the control. Analyses for protein–protein interactions and gene ontology (GO) were performed using String (http://string-db.org)^16^. GO by String was used to classify differentially expressed proteins according to functional enrichment.

### Enzyme-linked immunosorbent assay validation and statistical analysis

The analysis of the samples was performed using commercially available enzyme-linked immunosorbent assay (ELISA) kits, according to the manufacturer instructions. The following ELISA kits were used: transforming growth factor-β-induced protein ig-h3 (TGFBI; MyBioSource, San Diego, CA, USA; Catalogue No. #MBS177286; Lot No. #7481574715), cartilage oligomeric matrix protein/thrombospondin 5 (COMP; Merck Millipore, Saint Louis, MO, USA; Catalogue No. #1764; Lot No. #0102F2396), and angiotensinogen (AGT; Merck Millipore, Saint Louis, MO, USA; Catalogue No. #RAB1021; Lot No. #9217F2027).

The ROUT method was used for the outlier analysis. If outliers were identified, they were not included in the analysis. The dataset without outliers was tested for normality using Shaphiro–Wilk tests. To compare groups, if the data were normal (p > 0.05), parametric unpaired t-tests were used; if the data were not normal (p < 0.05), non-parametric Mann–Whitney tests were used. Statistical analysis was performed using GraphPad Prism 8. The level of significance was set at p < 0.05.

The linear support vector machine (SVM) classification model using selected proteins as features was trained and tested using stratified fivefold cross validation. Hyperparameters were selected using fivefold cross validation separately on the training sets of each split. The ROC curve and AUC calculation were based on the test sample predictions of each respective split.

### Ethics approval and consent to participate

The study was conducted with the approval of the Medical Ethics Committee of the Republic of Slovenia (No 0120-049/2016-4) and the research was performed in accordance with relevant regulations. The informed consent was obtained from all of the participants before their inclusion in the study. The research was carried out according to The Code of Ethics of the World Medical Association (Declaration of Helsinki). Trial registration number: NCT04591548.

## Results

### Characteristics of patients with endometriosis and control patients

The clinical characteristics of the patients with endometriosis (cases) and the control patients (controls) are presented in Table 1 for the discovery phase and in Table 2 for the validation phase. There were no significant differences between the case and control groups for either phase and for any of the characteristics examined here (Tables 1, 2, Supplementary Table S1).

#### Discovery phase

The mean age of the patients in the endometriosis group was 28.1 ± 3.1 years, and for the control group, 29.6 ± 2.8 years. All of these women had a BMI in the normal range (cases: 23.8 ± 1.7 kg/m^2^; controls: 23.8 ± 3.1 kg/m^2^) and a regular menstrual cycle (21–35 days). All of the samples were collected in the follicular phase of their menstrual cycle. All of the cases had endometriosis stage III, according to the Revised American Society for Reproductive Medicine classification of endometriosis^17^.

#### Validation phase

The mean age of the patients in the validation endometriosis group was 29.4 ± 3.5 years, and for the control group, 30.5 ± 4.3 years. All of these women had a BMI in the normal range (endometriosis group: 22.7 ± 3.7 kg/m^2^; control group: 21.8 ± 1.7 kg/m^2^), and a regular menstrual cycle (21–35 days). In the endometriosis group, 58% of samples were collected in the follicular phase of the menstrual cycle, and in the control group, 35%. The remaining samples were collected in the luteal phase of the menstrual cycle. There were no significant differences between the validation case and control groups for any of the characteristics examined here (Table 2). According to the laparoscopy and histological verification 19% had peritoneal endometriosis, 31% ovarian endometriosis, 38% combined ovarian endometriosis with peritoneal lesions, and 12% deep infiltrating endometriosis. Seventy-three percent of the validation cases had at least stage III disease, according to the Revised American Society for Reproductive Medicine Classification of Endometriosis^17^.

### Discovery of 16 proteins with significantly different levels in peritoneal fluid allows separation of patients with endometriosis from controls

The antibody microarray based analysis of 1360 proteins identified 16 proteins with significantly higher levels in the peritoneal fluid in cases (patients with endometriosis) *versus* controls (Table 3). All 16 of these proteins showed > 1.5-fold differences in their levels in the peritoneal fluid for the cases *versus* controls. The six proteins; angiotensinogen (AGT); proinflammatory calcium binding protein S1000A8/9 (S10A8/9); scavenger receptor cysteine-rich type 1 protein M130 (C163A); transforming growth factor-β-induced protein ig-h3 (TGFBI); epidermal growth factor receptor (EGFR) and tissue inhibitor of metalloproteinase 1 (TIMP1) showed the strongest differences with fold changes > 2 (log FC > 1). To the best of our knowledge, AGT, TGFBI, cartilage oligomeric matrix protein/thrombospondin 5 (COMP) and angiopoietin-4 (ANGP4) have not previously been associated with endometriosis.

**Table 3:** Proteins with different levels in the peritoneal fluid from endometriosis patients *versus* control patients.

Protein	Protein abbreviation	logFC	adjustedp-value*	Uniprot identifier
**Angiotensinogen**	**AGT, ANGT**	**1.70**	**3.1e**−**03**	**P01019**
Proinflammatory calcium binding protein S100A8/9	S10A8/9	1.66	2.8e−04	P05109
Scavenger receptor cysteine-rich type 1 protein M130	C163A	1.43	5.2e−07	Q86VB7
**Transforming growth factor-β-induced protein ig-h3**	**TGFBI, BGH3**	**1.23**	**3.8e**−**03**	**Q15582**
Epidermal growth factor receptor	EGFR, HER1	1.13	1.1e−02	P00533
Tissue inhibitor of metalloproteinase 1	TIMP1	1.08	2.8e−04	P01033
Lumican	LUM	0.95	1.3e−03	P51884
**Cartilage oligomeric matrix protein/thrombospondin 5**	**COMP**	**0.84**	**6.3e**−**04**	**P49747**
α-2-Antiplasmin	A2AP, SERPINF2	0.77	4.8e−02	P08697
Phospholipid hydroperoxide glutathione peroxidase	GPX4	0.72	3.2e−02	P36969
Insulin-like growth factor-binding protein 4	IBP4, IGFBP4	0.68	4.8e−02	P22692
Hepatocyte growth factor activator	HGFA	0.68	3.2e−02	Q04756
Matrix metalloproteinase-2	MMP2	0.66	8.2e−03	P08253
Dickkopf-related protein 3	DKK3	0.60	2.7e−03	Q9UBP4
Cellular tumour antigen p53	P53	0.59	1.1e−02	P04637
Angiopoietin-4	ANGP4	0.53	4.7e−02	Q9Y264

The proteins given in bold were selected for the validation study.

*p values adjusted for multiple testing according to Bonferoni and Hochberg.

**Figure 2:**
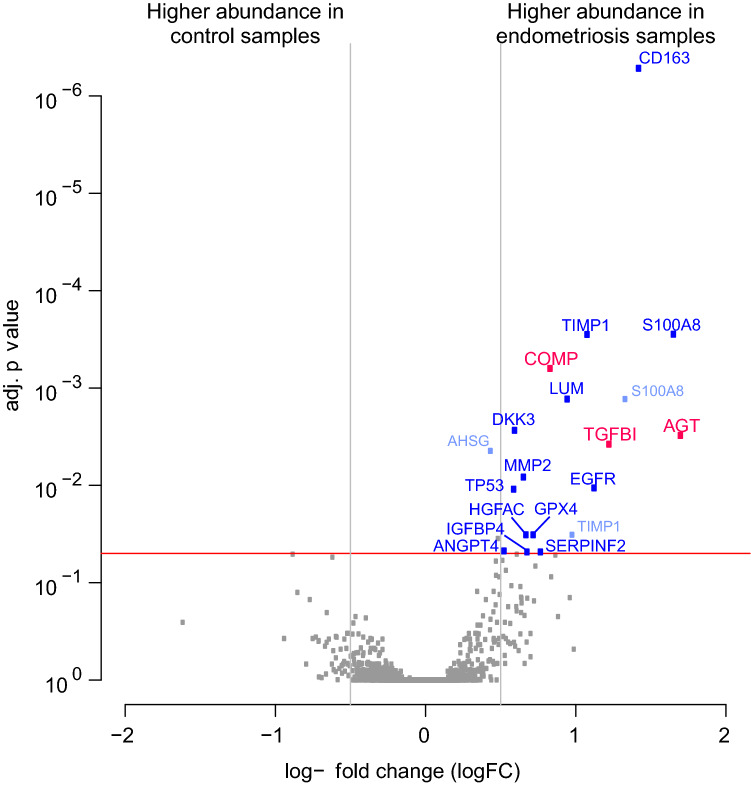
Volcano plot of the protein array data to visualise the adjusted p values and the corresponding log-fold changes (logFC). Horizontal red line, adjusted p = 0.05; vertical lines, logFC cut-offs (IlogFCI > 0.5). Proteins with positive logFC had higher levels in the peritoneal fluid of the cases *versus* controls; and vice versa for proteins with negative logFC.

The results of the statistical analysis of these protein array data are presented as volcano plots in Fig. 2. For these plots, the proteins with significantly higher levels in the peritoneal fluid of cases *versus* controls are positioned on the right side, above the red line that indicates the significance level of adjusted p value < 0.05.

**Figure 3:**
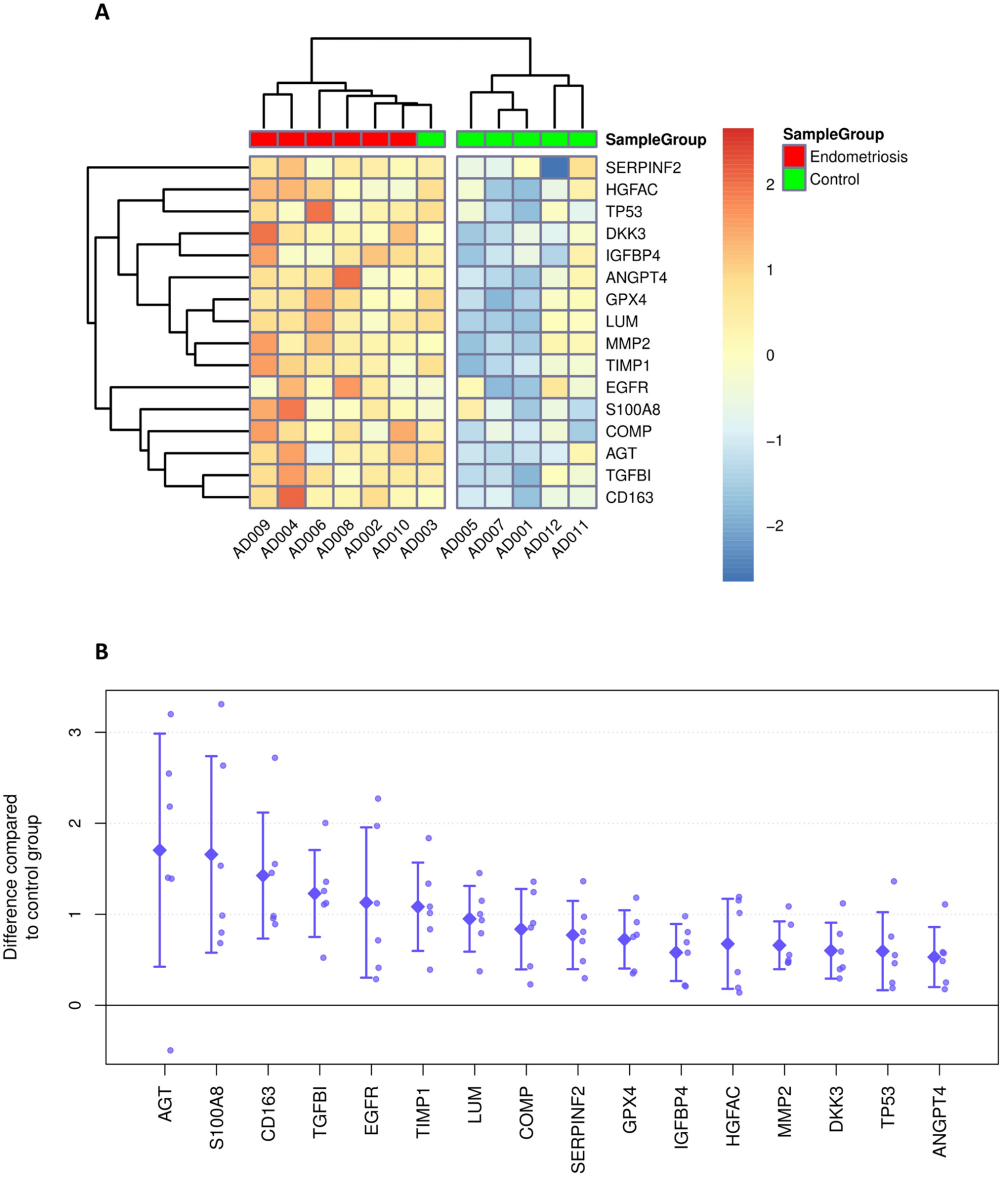
Heatmap displaying the relative expression of proteins identified as differential. Values were centered and scaled by proteins (**A**). Array value differences between individual endometriosis samples and the average of control samples for the selected differential proteins (**B**). Rhombs indicate sample group means. Whiskers indicate one standard deviation.

Hierarchical clustering of the array data filtered for these 16 differential proteins nicely separated patients with endometriosis from control patients (Fig. 3). Only the AD003 control sample clustered together with endometriosis samples, although there were no apparent reasons for this.

### Validation confirms higher levels of COMP and TGFBI in peritoneal fluid from patients with endometriosis versus controls

**Figure 4:**
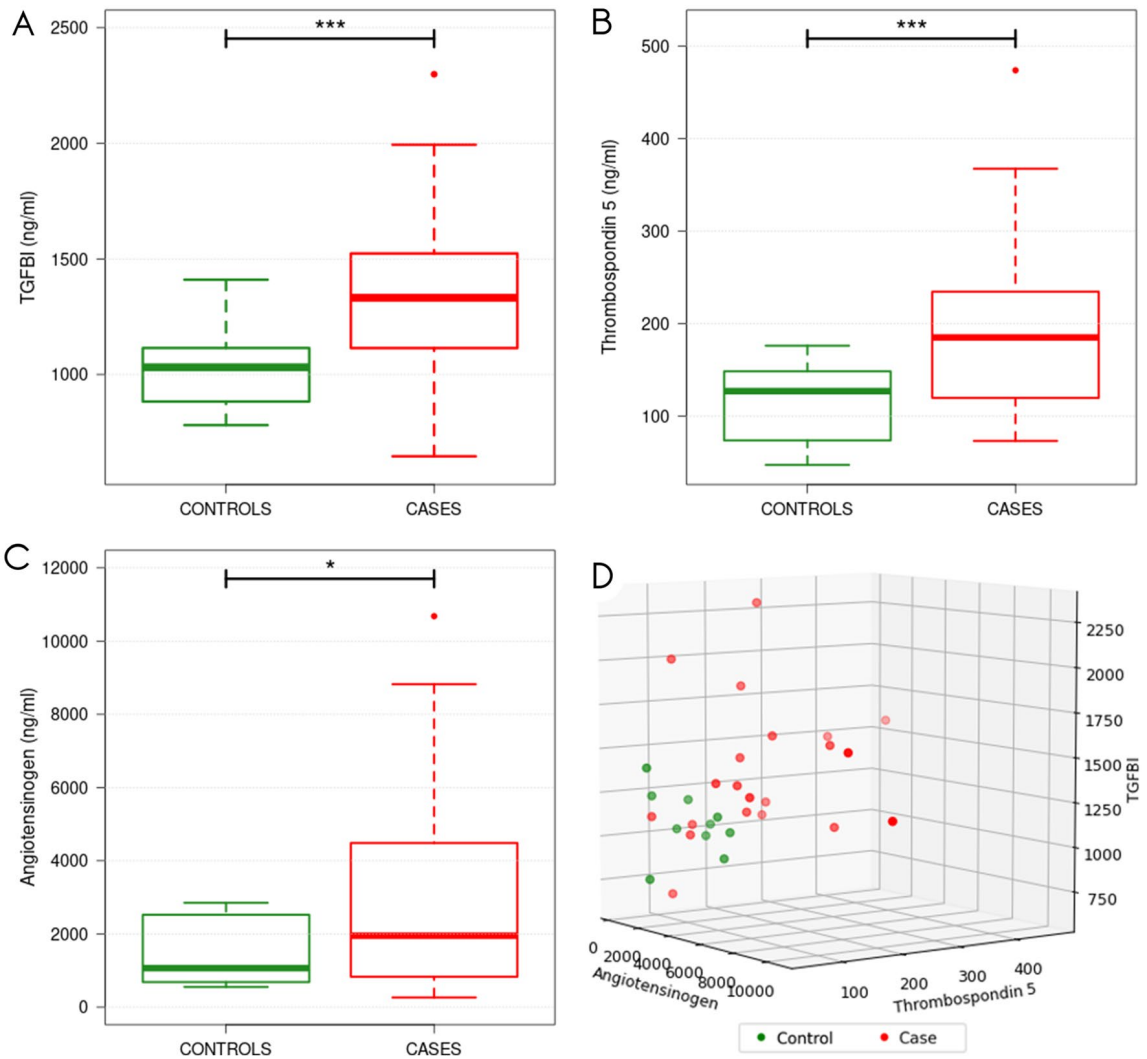
Validation of transforming growth factor-β–induced protein ig-h3 (TGFBI) (**A**), cartilage oligomeric matrix protein/thrombospondin 5 (COMP) (**B**) and angiotensinogen (AGT) (**C**)  levels in peritoneal fluid of endometriosis patients and control patients. 3D Scatterplot (**D**) shows distribution of TGFBI, cartilage oligomeric matrix protein/thrombospondin 5 (COMP) and angiotensinogen (AGT) levels across samples with measurements for all three proteins.

Three proteins that had not been associated with endometriosis previously were selected for validation using ELISA. This validation confirmed that the levels of the COMP and TGFBI proteins in the peritoneal fluid of cases *versus* controls were consistent with the microarray proteomic discovery study (Fig. 4). These COMP and TGFBI levels were significantly higher, at 1.7-fold and 1.3-fold for cases *versus* controls, respectively (p < 0.0005, for both). The levels of AGT were also 1.9-fold higher in the endometriosis patients *versus* the control patients (p = 0.0199).

**Figure 5:**
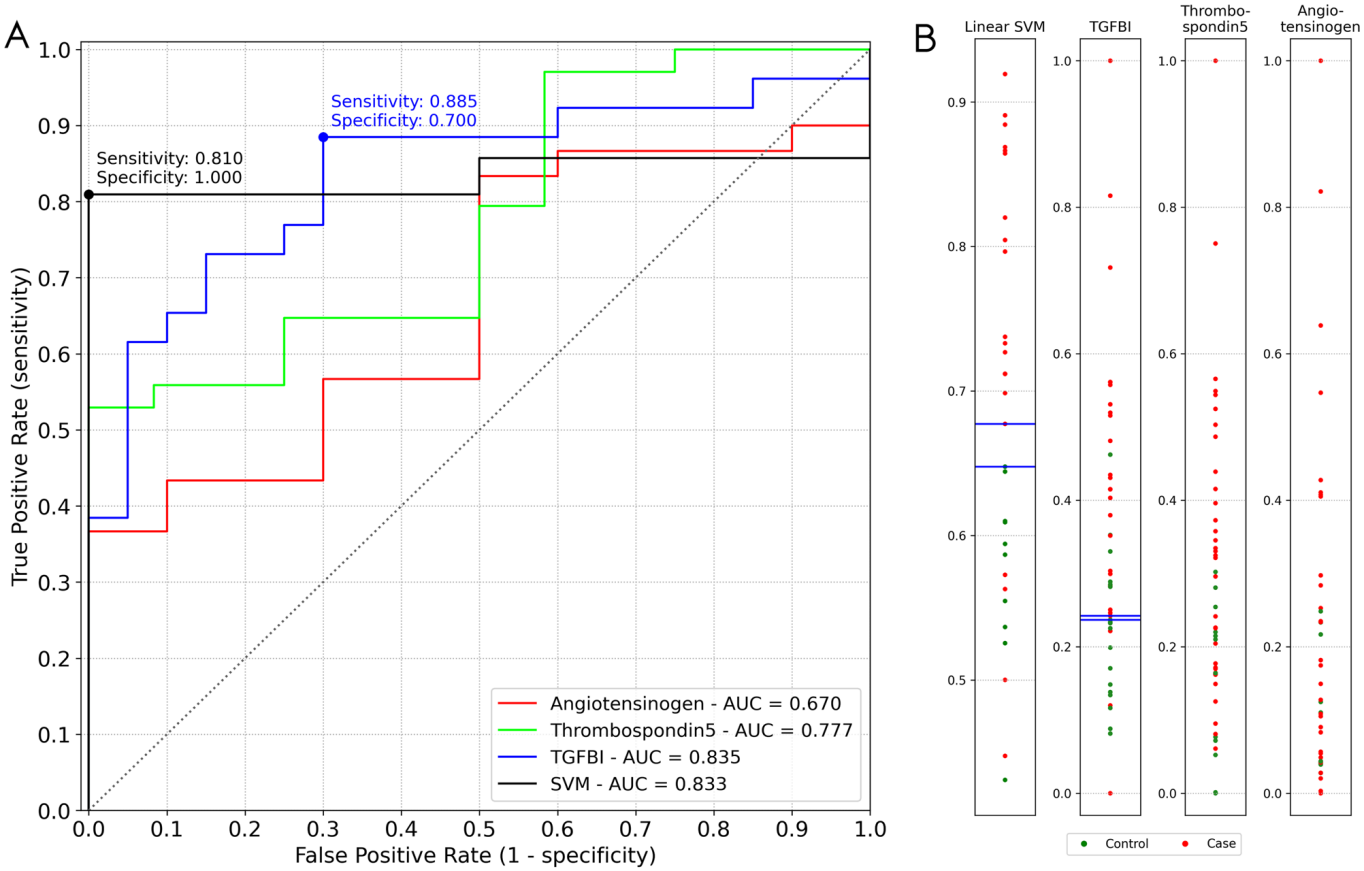
ROC curves assessing the diagnostic profiles of ig-h3 (TGFBI), thrombospondin 5 (COMP) and angiotensinogen (AGT) and a linear SVM model using all three features. Dots represent the decision thresholds yielding the most promising classifiers for the TGFBI and SVM predictors (**A**). One-dimensional sample distributions. For the single feature distributions, values were [0,1]-transformed. For the SVM distribution, calculated class probabilities for the class "case" were plotted (**B**). Blue lines mark samples for each group which are closest to the respective optimal decision thresholds of (**A**).

Further receiver operating characteristic (ROC) analysis revealed that COMP and TGFBI have very good diagnostic characteristics, with areas under the curve of 0.78 and 0.84, respectively (Fig. 5). With the cut-off point selected nearest to the top left-most corner of the ROC curve^18^, as a cut-off of 1047 ng/mL, TGFBI showed sensitivity of 88.5% and specificity of 70.0%, and with a cut-off of 180 ng/mL, COMP sensitivity was 95.0% and specificity was 54.3%. AGT showed only weak diagnostic potential, with an area under the curve of 0.67. An additional classification model based on a linear support vector machine (SVM) using all three proteins was generated, yielding an AUC of > 0.83, as well as sensitivity of 0.81 and a specificity of 1.00 at the optimal classification cutoff. An investigation of the sample distributions (Fig. 5B) reveals that, in terms of TGFBI values, at the chosen cutoff there is low separation between control and endometriosis samples, meaning a classifier based on TGFBI could potentially be sensitive to slight measurement variations. Comparatively, the margin between sample groups is more robust when applying the SVM classifier.

### Protein–protein interactions and gene ontology analysis identify direct and indirect interactions between these proteins that show differential levels in peritoneal fluid

**Figure 6:**
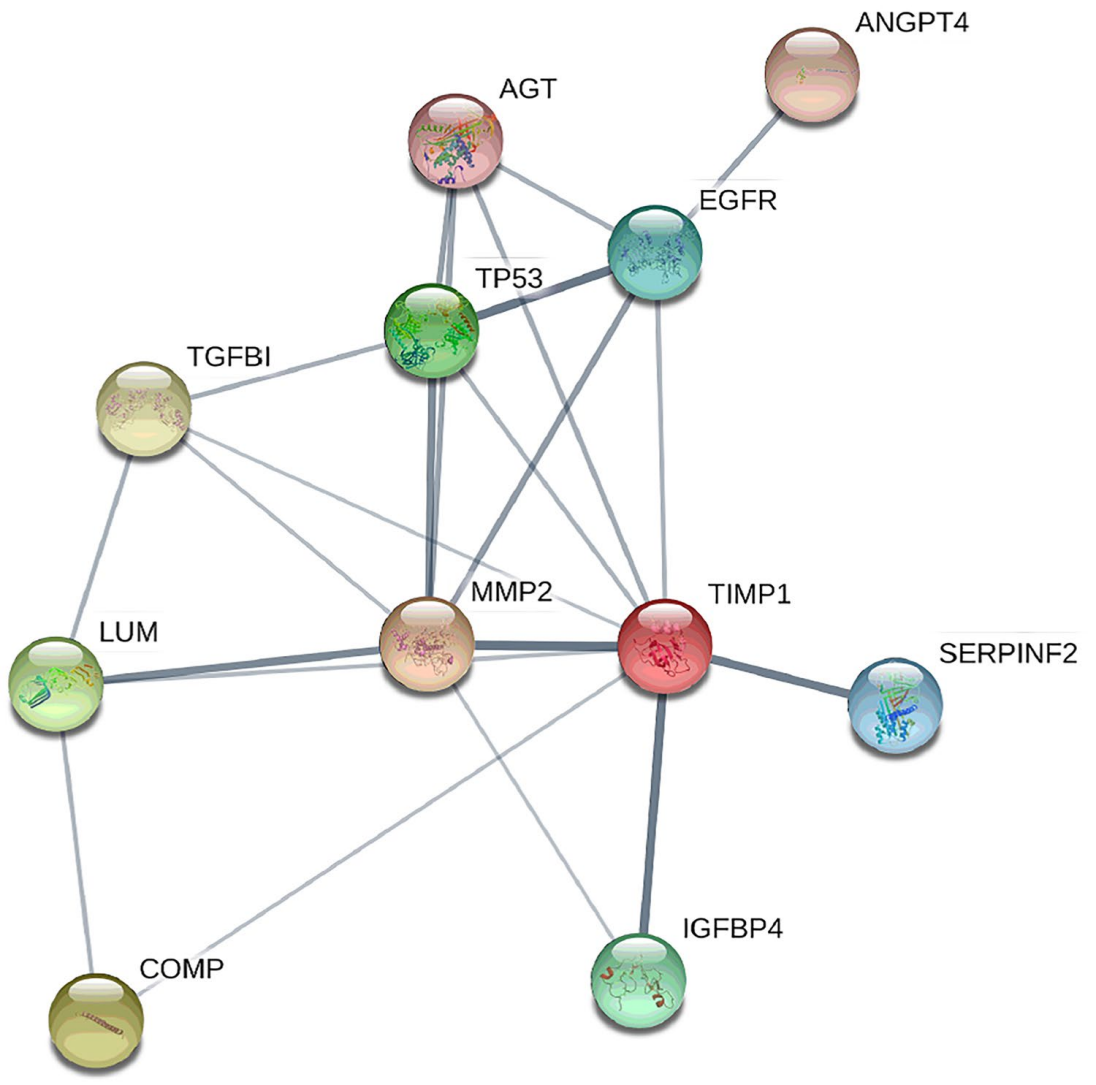
Analysis of protein–protein interactions from the STRING database for association networks. These revealed several direct (thick lines) and indirect (thin lines) interactions of the biomarker candidates. See Table 3 for protein name abbreviations.

GO analysis of differentially abundant proteins revealed that among molecular functions; signaling receptor binding, enzyme binding, protein-containing complex binding, protease binding, collagen binding and endopeptidase inhibitor activity were most enriched (Supplementary Table S2). KEGG pathways related to the differential proteins include Proteoglycans in cancer, PI3K-Akt, HIF-1 and MAPK signaling pathways, Bladder cancer, Endocrine resistance, Human papilloma virus infection, Pathways in cancer and others (Supplementary Table S3). The protein–protein interaction analysis using the STRING database^16^ revealed several direct and indirect interactions between the biomarker candidates (Fig. 6). Most of the proteins identified (i.e., MMP2, TIMP1, COMP, EGFR, AGT, ANGP4, TGFBI, HGFA, SERPINF2, GPX4, IGFBP4) are located in the extracellular region and are mainly involved in extracellular matrix re-modulation, negative regulation of apoptosis, inflammatory responses and responses to stress.

## Discussion

Biomarker discovery in the field of endometriosis has so far focused mainly on biomarkers from blood^4,6,9,19–21^. Thus, studies on peritoneal fluid biomarkers remain rare^4,7^, and to the best of our knowledge, to date there have not been any proteomic antibody microarray analyses of peritoneal fluid in population of women with endometriosis. For diagnostic procedures, peritoneal fluid samples could not replace blood samples since the collection is more invasive and thus risky. However, studies of peritoneal fluid might contribute to the identification of blood biomarkers for non-invasive diagnosis of endometriosis considering the surface of the peritoneal cavity is large, and it allows passive dialysis of substances between the blood plasma and the peritoneal fluid^11–13^. Dorien et al. studied plasma samples from patients with endometriosis and a control group using an antibody multiplex array approach, although they concluded that discovery and verification of potential markers is challenging using this method, mainly due to issues of reproducibility^22^. The only protein identified as a potential biomarker was interleukin-31^22^.

Identification of potential biomarkers in peritoneal fluid, which represents the local environment of endometriotic lesions, might represent the first step towards identification of clinically important biomarkers for non-invasive diagnostics from other body fluids (e.g., peripheral blood, urine, saliva). Peritoneal fluid undoubtedly has a role in the etiopathogenesis of endometriotic lesions, and thus proteomic analysis might also provide new knowledge about the basic mechanisms of this disease.

The antibody microarray analysis that was used in the present study identified 16 proteins that showed differences, whereby their levels were all increased in peritoneal fluid from patients with endometriosis (cases) compared to the control patients. Six of these proteins showed the greatest increases in their levels, which were twofold to fourfold higher for cases *versus* controls: AGT, S10A8/9, C163A, TGFBI, EGFR and TIMP1. According to our review of the literature here, COMP, AGT, TGFBI and ANGP4 have not yet been associated with endometriosis, while S100A8, C163A, EGFR and TIMP1 have^23–34^. However, EGFR has not yet been studied in peritoneal fluid, and for C163A, there is as yet no explanation of how it might be involved in the pathogenesis of this disease, with no data available on its potential value as a biomarker^25,26^. The other nine of these 16 proteins also showed statistically significant increased levels in the peritoneal fluid of the cases, which were ≥ 1.5-fold higher than for the controls.

For three candidate biomarkers, COMP, TGFBI and AGT, with increased levels in the peritoneal fluid no earlier reports in the context of endometriosis were found. Therefore, these proteins were selected for validation by ELISA. This validation confirmed the significant increases for COMP and TGFBI while the increase for AGT did not reach statistical significance.

COMP is a glycoprotein that is mainly localized to the extracellular matrix of cartilage, synovium, ligaments and tendons^35^. Increased levels of COMP have been associated with fibrogenesis in systemic sclerosis, skin keloids, vascular atherosclerosis, lung fibrosis, rheumatoid arthritis, osteoarthritis, pseudoachondroplasia, acute trauma and systemic lupus erythematosus^36^. In-vitro, COMP modulates pathological collagen-I deposition, despite up-regulation of matrix metalloproteinases^36^. We hypothesize that COMP induces collagen deposition and participates in extracellular matrix remodeling, and might thus contribute to the pathophysiology of intraperitoneal adhesions in endometriosis. Significantly higher levels of COMP in patients with endometriosis indicate that COMP has a role in pathogenesis of endometriosis and can serve as a potential biomarker or drug target.

TGFBI has been associated with a range of diseases, which include nephropathy, atherosclerosis, rheumatoid arthritis, corneal disorders and malignant diseases^37^. In malignant diseases, TGFBI appears to have either tumor-suppressing or tumor-promoting roles, with reports that suggest that TGFBI can mediate cancer cell invasion and metastasis, and can enhance cancer cell extravasation^38–41^. In ovarian cancer, TGFBI can enhance cell adhesion, motility and invasion^38^. The loss of TGFBI in cancer cells has a pro-tumorigenic role, while its overexpression in peritoneal cells aids the metastatic process^38^. As suggested for ovarian cancer, TGFBI might have a similar role in the development of endometriotic peritoneal implants.

These significantly higher levels of TGFBI in the peritoneal fluid from women with endometriosis in the present study suggest a role for TGFBI in the pathogenesis of endometriosis. This is the first report that has associated TGFBI with endometriosis, and we show the great potential for TGFBI as a diagnostic tool, and even more importantly, as a predictive biomarker for endometriosis. These data call for further validation studies, especially in patients with peritoneal endometriosis who are in great need for novel diagnostic options.

AGT is a component of the renin–angiotensin system. AGT is released from the liver and is cleaved in the peripheral blood by renin, to form angiotensin I. Angiotensin I is then converted into angiotensin II by angiotensin-converting enzyme. Angiotensin II is the most active component of the renin–angiotensin system, and it acts through interactions with two major receptors: the angiotensin II type 1 and type 2 receptors. One of the effects of activation of these receptors is stimulation of the synthesis of vascular endothelial growth factors, which can directly induce the formation of new blood vessels^42^. To date, only one study has reported any association of AGT with endometriosis, whereby Kowalczyńska et al. suggested a role for AGT M235T polymorphism in endometriosis. However, they did not provide any evidence for association of AGT with the development or clinical course of endometriosis, and did not indicate any prognostic value for AGT^43^. Although much remains to be learned, ANGT might have a role in the development and survival of endometriotic lesions in the peritoneal cavity, due to its biological function in angiogenesis.

The other proteins that were identified as showing specific changes in their levels in peritoneal fluid in endometriosis in our antibody array discovery analysis were MMP2, TIMP1, EGFR, ANGP4, C163A, HGFA, S10A8/9, LUM, A2AP/SERPINF2, GPX4, IBP4/IGFBP4, DKK3 and P53. These have not (yet) been validated as having important roles in endometriosis.

However, the expression of matrix metalloproteinases (MMPs) and the tissue inhibitors of metalloproteinases (TIMPs) have been shown to be involved in the pathogenesis and pathophysiology of endometriosis^44–49^. These proteins are at the forefront of extracellular matrix remodeling, and so changes in their levels might contribute to fibrosis and adhesions. A balance of these proteins is required for normal follicular development, ovulation, embryo implantation and further embryogenesis^50,51^. TIMP1 is secreted by endometriotic lesions, and it regulates cell differentiation, migration and death. Indeed, it might be part of the mechanism that causes endometriosis-associated infertility^52^. Significantly higher levels of TIMP-1 were found in peritoneal fluid of patients with endometriosis as compared to control women^28^. As shown in animal models, excessive TIMP1 was deleterious to ovulation and embryo development^52^. In the present study, the patients with endometriosis showed higher levels of TIMP1 and MMP2 in the peritoneal fluid, as compared to the controls. Protein–protein interaction analyses of the identified proteins have also confirmed the central position of MMP2 and TIMP1 in protein–protein interaction networks. Furthermore, TIMP1 has already been described as a protein that is secreted by endometriotic lesions^53^, and the present study supports its great value as a potential biomarker.

EGFR has important roles in signal-transduction, and it mediates a variety of cellular processes, including cell survival, proliferation, migration and angiogenesis, and inhibition of apoptosis^30,33^. It has a role in the cyclical growth of the endometrium, and might have a key role in the pathogenesis of endometriosis. Ejskjaer et al. have reported on different cyclical mRNA levels of EGF receptors and their ligands in eutopic endometrium from patients with endometriosis compared to endometrium from healthy individuals^34^, and EGF receptors in endometrium were significantly up-regulated in endometrial cancer and endometrial hyperplasia, compared to healthy menopausal endometrium^54,55^. EGFR is important also for female reproductive functions. Ding et al. reported higher levels of embryonic EGF and their receptors when mouse oocytes and embryos were cultured in media with peritoneal fluid obtained from women with mild endometriosis. Here, the fertilization of oocytes and the development potential of embryos were decreased^56^. In endometriosis most studies have compared *EGFR/HER1* expression in eutopic and ectopic endometrium, and have reported contradictory data: no significant differences^29–31^, and lower levels^32^ or higher EGFR protein and mRNA levels in eutopic and ectopic endometrium *versus* healthy endometrium^33,34^. In serum samples Matalliotakis et al. found no significant differences in the levels of soluble EGFR in patients with endometriosis *versus* those without endometriosis^57^. To the best of our knowledge, EGFR has not been studied in peritoneal fluid of women with endometriosis, where the higher levels in the present study suggest that it is association with subfertility.

Our antibody microarray proteomic analyses of the peritoneal fluid of patients with endometriosis also identified higher levels of angiopoetin 4 (ANGP4), a protein that is involved in angiogenesis. We have so far been unable to find any report of the detection of angiopoietin in peritoneal fluid, and also no reports about detection of angiopoietin in other body fluids in endometriosis patients. Angiopoietins have crucial roles through their promotion of pericyte recruitment and vascular branching^58^, and they might be involved in the pathogenesis of endometriosis, and thus might also represent potential biomarkers.

C163A has previously been investigated in serum and peritoneal fluid of women with endometriosis, but the data have been inconclusive^25–27^. C163A is a membrane receptor that is only expressed by monocytes and macrophages. The importance of peritoneal macrophages in the development of endometriosis is well known^59–61^. C163 has been reported to be related to the binding of hemoglobin:haptoglobin complexes^62^. C163A is regulated by other cytokines, where IL-6 and IL-10 have been shown to induce the expression of both its messenger RNA and the C163A surface receptor protein^63^. The extracellular (soluble) portion of this C163A (known as sC163) is shed from the cell surface when macrophages are stimulated by inflammatory cytokines^64^. The biological function of sC163 remains unknown to date, although it has been suggested to be a marker for monocyte/ macrophage activity in diverse inflammatory diseases^64,65^. There has only been one study so far that has examined sC163 in peritoneal fluid of women with endometriosis, and it reported no significant differences in comparison with the case group of patients who underwent laparoscopy due to infertility or elective tubal sterilization^27^. As we found higher levels of C163A in the peritoneal fluid of these women with endometriosis *versus* the controls, this indicates that C163 has some diagnostic potential.

Hepatocyte growth factor (HGF) is produced by endometrial stromal cells, and it promotes cell proliferation and migration, and lumen formation of endometrial epithelial cells^66,67^. Yoshida et al. showed significantly higher levels of HGF in peritoneal fluid of patients with endometriosis compared with patients without endometriosis^67^. Here, we found higher levels of one of its activators, hepatocyte growth factor activator (HGFA), which is consistent with the published literature on a role for the HGF system in the etiopathogenesis of endometriosis^67^.

Endometriosis is considered to be a chronic inflammatory disease, and previous studies have shown that inflammatory processes are involved in its pathogenesis and are associated with its characteristic symptoms. The pro-inflammatory calcium binding protein S100A8 has already been studied in patients with endometriosis, in samples of peritoneal fluid and cervical mucus^23,24^. S100A8 predominantly acts as a heterodimer with S100A9, and is thus named calprotectin. Overexpression at sites of inflammation has been well established for S100A8, and also elevated serum levels have been reported for a variety of inflammatory diseases^68–70^. S100A8 is released by phagocytes and is a potent chemoattractant for neutrophils and monocytes both in vitro and in vivo^71^. Our report here of higher levels of S100A8 in peritoneal fluid of women with endometriosis thus supports the published data^23^. A previous study has also reported higher protein levels for S100A8 in peritoneal fluid from patients with deep endometriosis, as compared to patients with superficial lesions^23^. There is no literature on S100A8 in the peripheral blood of patients with endometriosis. As S100A8 is known to be involved in inflammatory processes, it is probably not specific to endometriosis, and subsequently, its diagnostic value will be limited.

## Conclusion

To the best of our knowledge, the present study is the first that has used antibody arrays for the identification of differential levels of proteins in peritoneal fluid from patients with endometriosis. We defined 16 proteins with significantly increased levels in this peritoneal fluid, which are mainly related to fibrinogenesis, extracellular remodeling, pathogenesis of inflammation, induction of a dysfunctional immune system, and angiogenesis. This study also reports the first time that the proteins COMP, AGT, TGFBI and ANGP4 have been associated with endometriosis. For COMP and TGFBI, validation by ELISA confirmed the proteomic array data obtained here. Our findings have brought new knowledge that will contribute to better understanding of the pathophysiology of endometriosis. COMP and TGFBI thus represent potential biomarkers, which therefore warrant further studies also in blood samples, which are currently in progress.

## Supplementary Information

Supplementary Information.

## Data Availability

The datasets used and/or analyzed during the current study are available from the corresponding author on reasonable request.
