# Supplementary material for: Proteomic analysis of peritoneal fluid identified COMP and TGFBI as new candidate biomarkers for endometriosis
Source: Sci Rep. 2021 Oct 22;11:20870. doi: 10.1038/s41598-021-00299-2 (PMC8536751; doi:10.1038/s41598-021-00299-2)
Supplement: Supplementary file 1 — Supplementary Information. [file 41598_2021_299_MOESM1_ESM.docx]

Supplementary Table 1. Life style data of the 46 patients included in the validation phase.

| **Parameter** | **Units** | **Detail** | **Controls** | **Cases** | **p-value^*^** |
| --- | --- | --- | --- | --- | --- |
| Alcohol use | n (%) | Never | 5 (25) | 10 (38.5) | 0.374 |
|  |  | Occasionally | 13 (65) | 14 (53.8) |  |
|  |  | Once a week | 2 (10) | 2 (7.7) |  |
|  |  | 2-3 times per week | 0 (0) | 0 (0) |  |
|  |  | More than 3 times per week | 0 (0) | 0 (0) |  |
| Sport/recreation | n (%) | Regularly | 8 (40) | 7 (26.9) | 0.644 |
|  |  | Occasionally | 5 (25) | 8 (30.8) |  |
|  |  | No recreation | 7 (35) | 11 (42.3) |  |
| Stress (not feeling in control) | n (%) | Never | 5 (25) | 7 (26.9) | 0.339 |
|  |  | Almost never | 11 (55) | 8 (30.8) |  |
|  |  | Sometimes | 3 (15) | 7 (26.9) |  |
|  |  | Quite often | 0 (0) | 3 (11.6) |  |
|  |  | Very often | 1 (5) | 1 (3.8) |  |
| Stress (sense of being in control) | n (%) | Never | 0 (0) | 0 (0) | 0.288 |
|  |  | Almost never | 1 (5) | 2 (7.7) |  |
|  |  | Sometimes | 4 (20) | 8 (30.8) |  |
|  |  | Quite often | 12 (60) | 14 (53.8) |  |
|  |  | Very often | 3 (15) | 2 (7.7) |  |
| Stress (life going in right direction) | n (%) | Never | 1 (5) | 1 (3.8) | 0.735 |
|  |  | Almost never | 1 (5) | 3 (11.5) |  |
|  |  | Sometimes | 6 (30) | 8 (30.8) |  |
|  |  | Quite often | 9 (45) | 10 (38.5) |  |
|  |  | Very often | 3 (15) | 4 (15.4) |  |
| Stress (unable to cope with problems) | n (%) | Never | 4 (20) | 4 (15.4) | 0.660 |
|  |  | Almost never | 6 (30) | 6 (23) |  |
|  |  | Sometimes | 6 (30) | 12 (46.2) |  |
|  |  | Quite often | 3 (15) | 2 (7.7) |  |
|  |  | Very often | 1 (5) | 2 (7.7) |  |

*Chi-square test for trend

**Supplementary Table 2:** Selected molecular functions related to proteins with differential abundance in Endometriosis vs Control.

**Supplementary Table 3:** Selected KEGG pathways related to proteins with differential abundance in Endometriosis vs Control.

Kanehisa, M. and Goto, S.; KEGG: Kyoto Encyclopedia of Genes and Genomes. Nucleic Acids Res. 28, 27-30 (2000).

Kanehisa, M; Toward understanding the origin and evolution of cellular organisms. Protein Sci. 28, 1947-1951 (2019)

Kanehisa, M., Furumichi, M., Sato, Y., Ishiguro-Watanabe, M., and Tanabe, M.; KEGG: integrating viruses and cellular organisms. Nucleic Acids Res. 49, D545-D551 (2021)
